# The Use of the Suboccipital Transtentorial Approach to the Posterior Inferior Incisural Space

**DOI:** 10.7759/cureus.47705

**Published:** 2023-10-26

**Authors:** Jarnail Bal, Rory J Fairhead, Samir Matloob, Jonathan Shapey, Rossana Romani, Cormac Gavin, Alireza Shoakazemi, Jonathan Pollock

**Affiliations:** 1 Neurosurgery, Royal London Hospital, London, GBR; 2 Neurosurgery, Queen's Hospital, London, GBR; 3 Neurosurgery, King's College London, London, GBR; 4 Neurosurgery, Southampton General Hospital National Health Service (NHS) Foundation Trust, London, GBR; 5 Neurosurgery, The Royal Hallamshire Hospital, Sheffield, GBR

**Keywords:** meningioma, cranial microsurgery, posterior cranial fossa tumor, posterior incisural space, suboccipital transtentorial

## Abstract

Objective

To describe our experience with the microsurgical technique of the suboccipital transtentorial (SOTT) approach in the removal of posterior fossa lesions located in the posterior incisural space.

Method

Between 2002 and 2020 we reviewed all patients who underwent microsurgical resection of lesions of the posterior incisural space at the Department of Neurosurgery, Essex Neuroscience Centre, London, England (eight patients, male to female 3:5, mean age: 51, range 35-69). We describe the preoperative symptoms, radiological findings, surgical techniques, histology and postoperative outcomes in this cohort of patients.

Results

Eight patients with tumours located in the posterior incisural space underwent surgery during the study period including four meningiomas (50%), two haemangioblastomas (25%), one metastasis (13%) and one giant prolactinoma (13%). Gross or near total resection was achieved in six patients (75%): the giant prolactinoma could not be radically removed and one of the meningiomas required a small fragment to be left in place to protect the Vein of Galen. No patient developed a visual field deficit due to occipital lobe retraction. One patient developed a temporary trochlear nerve palsy (13%). Five patients had mild disability (Glasgow Outcome Scale (GOS) = 5), and four had moderate disability (GOS = 4).

Conclusion

In our series, the SOTT approach provided excellent access for all cases of tumours in the posterior incisural space. The tumour’s size and relationship to the deep venous system contributed to the choice of approach and in one patient who had previously undergone surgery via the supracerebellar route, the SOTT approach enabled the avoidance of gliotic scar tissue. Success is dependent on careful case selection, though from our series of 8 patients, we conclude that this approach allows safe access to the posterior incisural space, with acceptable outcomes with regard to postoperative disability and cranial nerve palsy. As such, the approach should be in the armamentarium of any neurosurgeon who regularly deals with posterior fossa pathology.

## Introduction

The microsurgical approach to lesions originating from the posterolateral brainstem, tectal plate area and tentorial incisura space requires the application of careful decision-making to select the optimum surgical approach [1]. As with all procedures requiring relatively deep intracranial access, important considerations are (1) the need for maximum relaxation of the neighbouring brain to minimise morbidity from brain retraction; (2) the need to identify, protect and safely separate the surrounding neurovascular structures, especially cranial nerves and deep cerebral veins, without injury from the target surgical lesion; and (3) application of detailed anatomical knowledge, aided by surgical morphometric studies, to delineate anatomy [2,3]. The posterior incisural space is located posterior to the midbrain and the pineal region [4]. The most common lesions found in this region in adults are meningiomas arising from the tentorium, superior clivus or petrous apex, pineal tumours, metastases, haemangioblastomas of the cerebellum and vascular malformations [4]. Surgical approaches described include the supracerebellar infratentorial approach, the occipital transtentorial approach, and combined supra-infratentorial approaches [1,5-7]. Optimal neuroanesthesthetic care with particular care in optimising pCO2 that is maintaining a pCO2 below 4.5 KPa is mandatory to produce brain relaxation during the approach to this region. This is valid for all approaches to this deep region to avoid excess brain retraction [4].

In the present report, we retrospectively report eight consecutive patients with posterior incisural space lesions operated on via the suboccipital transtentorial (SOTT) approach by the senior author (JRP) between January 2002 and December 2019 at the Department of Neurosurgery, Essex Neuroscience Center, London, England. To date, there have been few studies describing patient outcomes through this specific approach. In this paper, we hope to educate on the anatomy and technical considerations of the approach and we demonstrate that the SOTT approach was found to be safe and satisfactory for the removal of this series of lesions in this location.

## Materials and methods

Case series

Between 2002 and 2020 eight consecutive patients with posterior incisural space lesions were selected for the SOTT approach by the senior author (JRP) (male to female 3:5, mean age: 51, range 35-69). Lesions selected for this approach were either situated immediately inferior to the leading edge of the tentorium or partly above and partly below the tentorium. The origin of the tumour approach included the tentorium itself, the petrous or superior clival surface and the cortex of the cerebellum. All patients underwent preoperative MRI and seven patients also had a preoperative CT scan. All cases were discussed in a skull base multidisciplinary team (MDT) meeting prior to surgical intervention. Clinical performance status after surgery was expressed with the Glasgow Outcome Scale (GOS)[8]. MRI was used to record the postoperative radiological outcome.

Anatomical considerations of the posterior incisural space

Neural and Cisternal Structures

The posterior incisural space is situated behind the midbrain and includes the pineal region. The roof of this anatomical region is formed by the lower surface of the splenium, the crura of the fornices and the hippocampal commissure; the floor is formed by the superior surface of the vermis (divided by short deep fissures: lingual, central lobule, culmen and declive) and the anterior wall is formed by the posterior third ventricle, pineal body, and quadrigeminal plate. Below the colliculi, the anterior wall is formed in the midline by the lingula of the vermis and laterally by the superior cerebellar peduncle. The posteroinferior surface of the temporal lobe forms the lateral wall of the posterior incisural space. The tentorial apex is the posterior border of the posterior incisural space [4,9].

CSF Spaces

The main cistern of the posterior incisural space is the quadrigeminal cistern posterior to the quadrigeminal plate; this cistern communicates inferolaterally into the posterior part of the ambient cistern located between the midbrain and the parahippocampal gyrus [4,9].

Arterial Structures

There are branches from two main arteries running in the posterior incisural space: the second and third segments (P2 and P3 respectively) of the posterior cerebral artery (PCA) and superior cerebellar artery (SCA). The P2 segment extends from the posterior communicating artery to the entry point of the quadrigeminal cistern; P3 begins at the posterior midbrain and courses within the quadrigeminal cistern to end at the anterior limit of the calcarine fissure. P2 branches include the medial posterior choroidal artery which enters the posterior incisural space from anterior to posterior beside the pineal body and enters the velum interpositum; and the lateral choroidal artery which arises in the posterior incisural space passes around the posteromedial surface of the pulvinar and through the choroidal fissure. The SCA courses in the cerebellomesencephalic fissure to reach the posterior incisural space. Below the free edge of the tentorium, there are branches of the SCA which supply the tentorial surface of the cerebellum [9-11].

Venous Structures

The internal cerebral and the basal veins form the vein of Galen in the posterior incisural space. The vein of Galen passes below the splenium and enters the straight sinus at the tentorial apex. Several bridging veins from the cerebral hemisphere, cerebellum, tentorium and brainstem enter this system [2,4,11]. The presence of the midline vein of Galen is an important factor when selecting cases for surgery via the SOTT approach as very large centrally placed lesions in this location are not completely accessible through a unilateral approach [10,11].

Surgical technique

Induction of Anaesthesia, Positioning and Surgical Approach

Mannitol 20% i.v (1g/kg) and 8mg i.v. of dexamethasone were given to all patients at the induction of anaesthesia to achieve adequate brain relaxation. A total of 1.5g of i.v cefuroxime was administered to all patients according to our departmental antimicrobial protocol. A pCO2 in the range of 4.0kPa to 4.5kPa was maintained during the procedure with control of systolic blood pressure targeted towards a maximum systolic pressure of 100mmHg.

The patients were positioned in either a prone position on a Montreal mattress (six patients) or in a lateral position (three patients). In the case of a very extensive giant pituitary adenoma, the SOTT operation comprised the second stage of a two-stage debulking, the first stage having previously been done via the transsphenoidal route. The head was fixed in the three-pin skull clamp. Neuronavigation (Stealthstation, Medtronic Inc., MN, USA) was used to plan the location of the bone flap and to optimize the surgical trajectory in all patients.

**Figure 1:**
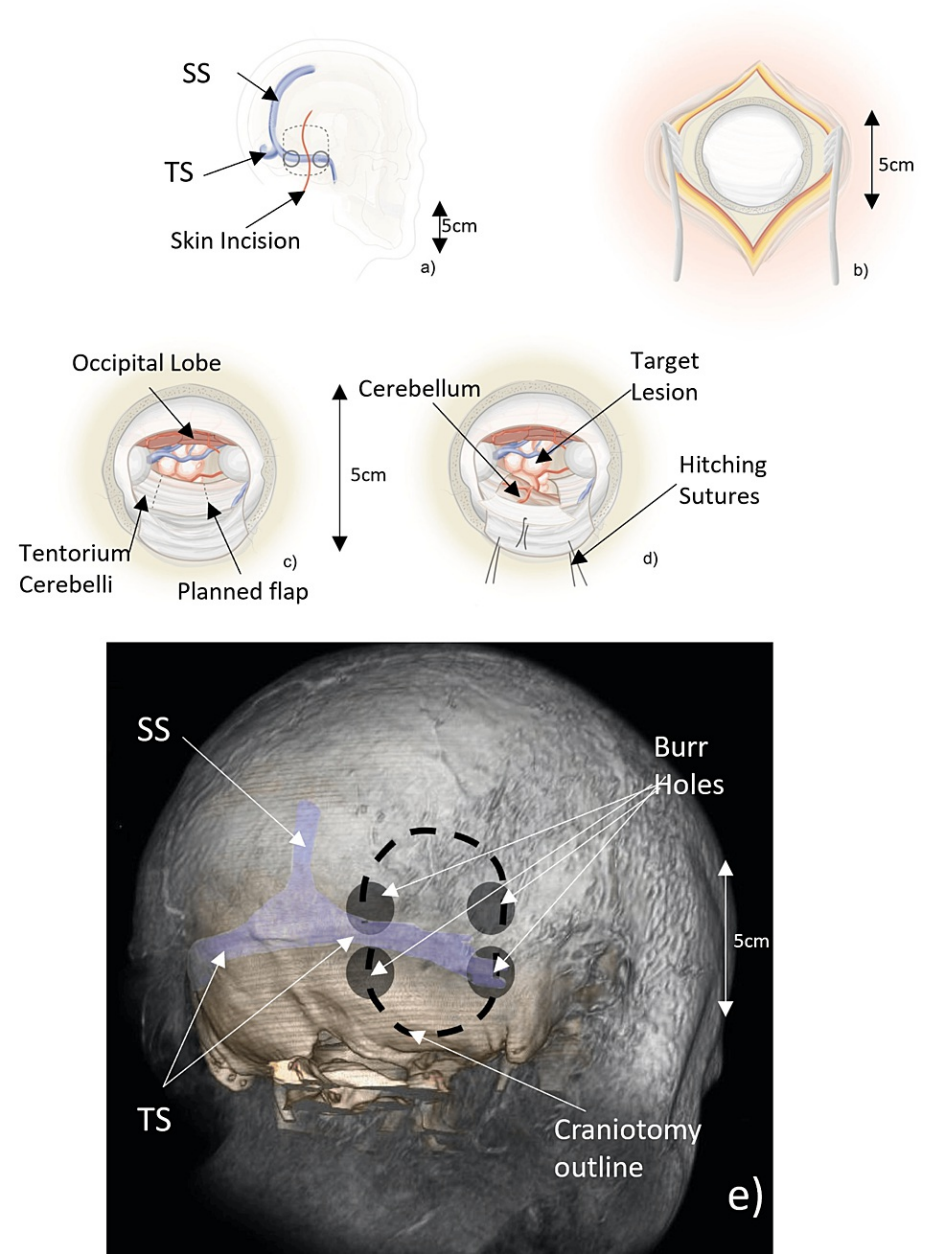
Steps in operative approach and craniotomy position in reference to vascular structures. Figures (a-d) as referenced in the text show steps in the SOTT operative approach and key structures encountered in the operative field. Figure (e) shows the position of burrholes and craniotomy in relation to important underlying vascular structures. TS - transverse sinus, SS - superior sagittal sinus, SOTT - suboccipital transtentorial Figure 1e courtesy: Mr Cormac Gavin

A curvilinear 10-12 cm paramedian skin incision perpendicular to the transverse sinus (TS) was used in all cases. The incision extended two-thirds above and one-third below the TS (Figure 1a). Care was taken to select an approach sufficiently medial in order to avoid traction on the inferior anastomotic vein of Labbè where it joins the transverse-sigmoid junction. The suboccipital muscles underlying the inferior third of the incision were incised parallel to their fibres with monopolar diathermy and separated in order to carry the incision down to the bone. A free bone flap of approximately 6x3 cm was cut using two burr holes based on the TS and a small deltoid craniotomy from these burrholes above the TS overlying the occipital dura (Figures 1b, 1e). The dura was elevated to expose the TS beneath the inferior part of the craniotomy. The relative lack of a deep groove for the TS is an anatomical feature of this region rendering bone elevation straightforward.

Dural Opening and Tumour Resection

A C-shaped durotomy based on the TS was made and the dura elevated. The dural flap was retained with hitch sutures. Occipital polar veins are usually few or absent [11], permitting ready access to the suboccipital subdural space via this approach. However, when discovered the occipital polar veins may be coagulated and divided with minimal morbidity. No major bridging veins between the occipital cortex and the TS were encountered in any patient in this series. The operating microscope was brought in and progressive and gentle elevation of the occipital lobe was performed to expose the tentorium. With appropriate neuroanaesthesia and optimisation of pCO2, exposure of the superior surface of the tentorial dura may be achieved with minimal gentle retraction. The surface of the occipital lobe was protected with patties made of low-adherence dressing material (Melolite®, Smith+Nephew Plc., UK) and the exposure was maintained using the single blade of a self-retaining retractor system. CSF was generously drained by sharp dissection of the arachnoid roofing over the basal cisterns. Image-guided aspiration of the occipital horn of the ipsilateral lateral ventricle may also be performed, especially if the target tumour has produced a degree of preoperative ventricular enlargement. The approach phase to the tentorial hiatus typically proceeded slowly to permit full relaxation of the occipital lobe without applying significant pressure to the occipital lobe.

With the use of image guidance to guide the trajectory of the tentorium overlying, the index lesion was exposed. A tentorial flap was planned and the margins coagulated with bipolar diathermy (Figure 1c). A box flap was incised in the tentorium; the leading edge of the tentorium forms the apex of the flap with two approximately perpendicular tentorial incisions defining the lateral and medial edges of the flap (Figure 1d). Care was taken to identify and protect the trochlear nerve by placing the anterior of the two tentorial incisions several millimetres behind the point where the nerve merges with the leading edge of the tentorium. The tentorial incision was tailored to the size of the underlying posterior fossa lesion. The maximum size of the leading edge of the flap available was determined by the distance between the point posterior to the trochlear nerve (as described above) and the most medial incision that could be made without incising the vein of Galen.

In cases where the tentorium comprised part of the tumour origin, the overlying tentorium was resected. This was also an option for other tumours where additional access was required. When the dural flap was not resected, a hitch stitch was placed in the tentorial edge and gentle traction was applied; the flap was retained beneath the single occipital retractor blade used to protect the access corridor with care taken to protect the trochlear nerve from any traction. The target tumour may then be removed using a standard microsurgical technique with the help of fine bipolar forceps and microsuction. A small cerebellar corticotomy or corticectomy may be required to expose and remove intrahemispheric lesions such as the haemangioblastoma described in this series. Arachnoid adhesions of the ambient and quadrigeminal cisterns were sharply dissected when needed to visualise the basal veins and the great vein of Galen both of which are carefully protected. A cavitron ultrasonic surgical aspirator such as the CUSA® (Integra LifeSciences, Princeton, USA) device may be used to debulk large tumour volumes though en-bloc resection was preferred for small lesions. Following tumour removal, meticulous haemostasis was performed using bipolar forceps and surgical wound closure was performed in layers in the usual manner.

The tentorial flap is replaced but not sutured. The dural opening is sutured in a watertight fashion with reinforcement of the repair with hydrogel sealant. The bone is fixed with multiple titanium miniplates. Multilayered muscle closure without a subgaleal drain is done prior to skin closure.

## Results

Each patient’s demographic data, preoperative symptoms, tumour details, surgical summary and clinical outcomes are listed in Table 1.

**Table 1:** Demographic, tumour, operative and outcome details of patients in the case series SOTT - suboccipital transtentorial, GOS - Glasgow Outcome Scale

Case	Age + sex	Presenting symptoms	Location	Max. diameter	Operative positioning	Surgical procedures	Excision	Outcome	Histology	GOS at 3 months
1	53F	Panhypopituitarism and hyperprolactinaemia 1. Acute visual decline requiring transsphenoidal approach 2. Subsequent cognitive decline, left-sided proptosis, drowsiness, impaired mobility, acute hemiparesis, acute onset of III, VI and VII cranial nerves palsies, Requiring second-stage surgery from a posterior approach.	Very extensive extra-prolactinoma arising from pituitary fossa, invading the left orbit, suprasellar region, anterior incisural space and posterior-incisural space	70mm	1. Supine 2. Prone	1. Urgent transsphenoidal approach 2. SOTT	1. Subtotal 2. Subtotal	Cabergoline started after first operation (Prolactin level 750000 mIU/l to normal) Improvement of visual acuity and hemiparesis	Prolactinoma with apoplexy	4
2	69M	Headache, dizziness, ataxia, right cerebellar syndrome	Right superior cerebellar cortex	28mm	1. Lateral 2. Lateral	1. Posterior fossa craniotomy 2. SOTT	1. Subtotal 2. Total	Mild ataxia persisting	Haemangioblastoma	5
3	63M	Ataxia, right cerebellar syndrome, hydrocephalus	Superior cerebellar vermis. extra-axial	30mm	Prone	SOTT	Subtotal	Mild ataxia, died 6 months later of multiple myeloma.	Meningioma WHO II	4
4	43F	Obstructive hydrocephalus, ataxia, cerebellar syndrome (Figure 2)	Right infratentorial extra-axial	45mm	Prone	1 Aug 2006: VP shunt 2 Feb 07: SOTT	Total	Right IV paresis, improvement of ataxia	Meningioma WHO I	5
5	35F	Refractory left trigeminal neuralgia, tingling sensation of the left-sided of the tongue (Figure 3)	Left petrous apex extra-axial	20mm	Lateral	SOTT	Total	Uncomplicated progress	Meningioma WHO II (Chordoid)	5
6	43F	Headache, ataxia, left facial palsy, impaired L corneal reflex	Superior cerebellar vermis	35mm	Prone	SOTT	Total	Uncomplicated progress	Metastatic breast carcinoma	4
7	54M	Headache, ataxia (Figure 4)	Left superior cerebellar cortex	28mm	Lateral	SOTT	Total	Uncomplicated progress	Haemangioblastoma	5
8	47F	Progressive right trigeminal sensory loss	Right lateral inferior tentorium	25mm	Prone	SOTT	Total	Transient trochlear paresis	Meningioma WHO I	5

Imaging and histology

Peritumoral vasogenic oedema was present in four patients. All patients had a postoperative MRI to confirm the extent of tumour removal (total vs. subtotal) the results of which are presented in Table 1. Definitive histology confirmed a meningioma in four patients (two WHO Grade I and two WHO Grade II), a haemangioblastoma in two patients, one prolactinoma and one breast metastasis.

Brain relaxation and CSF drainage

Optimum neuroanesthesia as described above and administration of IV mannitol was utilised in all cases. One patient (Case 4; Table 1) had prominent lateral ventricles preoperatively in the presence of a large meningioma of the tentorial edge. A temporary external ventricular drain was therefore placed following induction and removed two days after the procedure. In two patients (Case 2; Case 4), a lumbar drain following induction for the craniotomy and 50 mL of CSF was withdrawn during the approach phase of the procedure, and removed in the 24 h following the procedure. In all other cases, CSF was generously drained directly from the basal cisterns with a wide opening of the arachnoid of the cisterns as soon as this was accessible.

Tumour excision and postoperative complications

The extensive prolactinoma could not be radically removed and in one meningioma patient (Case 3), a fragment of meningioma closely applied to the vein of Galen prevented complete removal. One patient developed an ipsilateral trochlear paresis and diplopia which improved after subsequent treatment by an ophthalmic surgeon. Three patients had some degree of persistent mild ataxia at three months of follow-up.

Clinical outcomes

All patients made a good recovery from surgery. All patients underwent a postoperative MRI at three months after surgery which confirmed complete tumour resection in seven cases. There was no surgical mortality but the two patients with residual tumours died of unrelated causes. The prolactinoma patient suffered medical complications of her extensive hypopituitarism and died of respiratory complications 39 months after surgery. The patient with residual WHO II meningioma died of unrelated multiple myeloma six months following surgery and the patient with metastatic breast cancer died at 20 months. No tumour recurrence occurred during the study follow-up period and the remaining six patients remained well at the last recorded follow-up (median follow-up: seven years, range: two to nine).

## Discussion

Neoplastic lesions of the posterior incisural space, dorsolateral brainstem and anteromedial region of the superior cerebellum are uncommon and relatively inaccessible, requiring careful preoperative assessment to select the optimum approach for each case. Multiple surgical routes have been described to access these regions including midline, paramedian and extreme lateral supracerebellar infratentorial approaches [7,12-14], posterior subtemporal [15,16] occipital transtentorial [4,17], transpetrosal [6], combined supra- and infra-tentorial approaches [18] and occipital interhemispheric transtentorial [19]. The most frequently used approaches are the infratentorial supracerebellar (IS) and the SOTT [10,17,18,20].

Owing to the relative scarcity of posterior incisural space tumours and the multiple surgical approaches to the region, there is a paucity of literature describing the extent of resection and outcomes [21]. This paper presents one of the largest case series analysing patient outcomes and the extent of resection of patients undergoing surgery via the SOTT approach and the largest series from a UK centre to our knowledge [22]. Furthermore, there is considerable heterogeneity in surgical techniques within the SOTT approach [23], this study adds to the existing literature by presenting the techniques and knowledge gained through surgical experience from our centre, as well as by showing that the technique is safe, effective and has lead to minimal morbidity in our case series [24].

By utilising an approach to the posterior fossa from above via a separate dural compartment the SOTT may seem somewhat counter-intuitive. However, we have found this approach to be well-suited to these selected cases for several important reasons. The supratentorial compartment is much larger than the posterior fossa, which presents a greater compartmental volume in which to achieve relaxation of the brain compared with the relatively crowded space in the posterior fossa when adopting the supracerebellar infratentorial route. The basal cisterns can be freely accessed at the leading edge of the tentorium to facilitate generous CSF drainage. Whilst some retraction of the superior cortex of the cerebellum may be required during the SOTT for lesions lying in the inferior part of the posterior incisural space, this is far less than is required with the equivalent supracerebellar route [9]. The SOTT is therefore associated with a more direct and less obstructed trajectory from the occipital calvarial surface to the tumour than the equivalent supracerebellar approach. Quantitative analysis of exposure and manoeuvrability akin to studies on access to the nearby cisternal pulvinar (favouring a contralateral supracerebellar approach) [25] would be valuable next steps in refining the choice of approach.

Limitations of the SOTT approach

There are several aspects of this approach which require particular care. There is a reported visual deficit in 17% of patients [17,26-27] in association with the required occipital lobe retraction. Whilst we did not observe such a deficit in this series, the importance of optimum brain relaxation during the approach phase to allow adequate time for this to occur should be emphasised. A few minutes of extra time to permit CSF drainage during the approach pays dividends in the avoidance of unnecessarily forceful retraction during the procedure. In one case, we achieved additional relaxation by drainage of CSF from the ipsilateral occipital horn; performed without the need for a separate burrhole - a further advantage over infratentorial approaches (a technique originally described by Poppen in 1966) [1].

One patient suffered a temporary trochlear nerve palsy: a reminder of the vulnerability of this structure during this approach. However, it is arguable that in tumours lying at the edge of the tentorium, the trochlear nerve is better seen from above than would be achieved during an equivalent infratentorial approach when the nerve lies ‘uphill’.

Another limitation of this approach is that there is a chance that a significant venous lake may be encountered during the division of the tentorium. These are commonly described [28] but did not present a significant obstacle during these cases. Ligating clips should be available to deal with venous lakes during tentorial incisions. The neuroanaesthetist should always be informed when the tentorium is about to be incised because of the small risk of causing a venous air embolism.

When selecting the SOTT approach, the surgeon should be mindful of the limited access to the contralateral quadrigeminal region. Deep venous tributaries which cannot be sacrificed block part of this region when utilising a unilateral SOTT. A partial section of the splenium has been described to facilitate such access, but a bilateral approach is likely to be preferable when a large midline lesion in this region requires radical removal. A useful alternative when access to both the contralateral and ipsilateral quadrigeminal region is required has been described by Kawashima et al. [4]. This technique involves the same incision and craniotomy as the SOTT approach but division of the tentorium is done parallel to the straight sinus with the falx also incised above the straight sinus. The inferior tentorial sinus is sacrificed. It may be a challenge to locate the straight sinus and accurately map its dimensions to prevent injury to it in this technique. In very extensive lesions requiring combined infra- and supratentorial access, the traverse sinus can be sacrificed after appropriate investigation to establish non-dominance. In very extensive tumours a staged supra- and infra-tentorial access at separate sittings may be appropriate. Access to the posterior part of the third ventricle with safety is usually limited via the SOTT because of the extended depth of the approach [10]. The interhemispheric transcallosal transchoroidal route is likely to be a better choice in such cases [29]. This approach avoids the deep venous complex and may be a superior choice for smaller pineal region lesions which project anteriorly into the third ventricle and mandate a surgical approach.

## Conclusions

The SOTT approach as described here provided excellent access to the eight cases presented, with few complications and a good functional outcome. This approach offers a safe and satisfactory alternative to the supracerebellar route for many lesions of the posterior incisural space. Success is dependent on careful case selection but we consider that this approach should be in the armamentarium of any neurosurgeon who regularly deals with posterior fossa pathology.

## References

[1] Poppen JL (1966). The right occipital approach to a pinealoma. J Neurosurg.

[2] Akdag UB, Ogut E, Barut C (2020). Intraforaminal dural septations of the jugular foramen: a cadaveric study. World Neurosurg.

[3] Ogut E, Akdag UB, Kilincli MF, Barut C (2022). Reappraisal of the types of hypoglossal canal: endocranial approach. Anat Sci Int.

[4] Kawashima M, Rhoton AL Jr, Matsushima T (2002). Comparison of posterior approaches to the posterior incisural space: microsurgical anatomy and proposal of a new method, the occipital bi-transtentorial/falcine approach. Neurosurgery.

[5] Bruce JN, Stein BM (1995). Surgical management of pineal region tumors. Acta Neurochir (Wien).

[6] Cho CW, Al-Mefty O (2002). Combined petrosal approach to petroclival meningiomas. Neurosurgery.

[7] Stein BM (1971). The infratentorial supracerebellar approach to pineal lesions. J Neurosurg.

[8] Jennett B, Bond M (1975). Assessment of outcome after severe brain damage. Lancet.

[9] Rhoton AL Jr (2000). Tentorial incisura. Neurosurgery.

[10] Rhoton AL (2000). The posterior cranial fossa: microsurgical anatomy and surgical approaches. Neurosurgery.

[11] Rhoton AL Jr (2000). The posterior fossa veins. Neurosurgery.

[12] Laborde G, Gilsbach JM, Harders A, Seeger W (1992). Experience with the infratentorial supracerebellar approach in lesions of the quadrigeminal region, posterior third ventricle, culmen cerebelli, and cerebellar peduncle. Acta Neurochir (Wien).

[13] Ogata N, Yonekawa Y (1997). Paramedian supracerebellar approach to the upper brain stem and peduncular lesions. Neurosurgery.

[14] Vishteh AG, David CA, Marciano FF, Coscarella E, Spetzler RF (2000). Extreme lateral supracerebellar infratentorial approach to the posterolateral mesencephalon: technique and clinical experience. Neurosurgery.

[15] Smith ER, Chapman PH, Ogilvy CS (2003). Far posterior subtemporal approach to the dorsolateral brainstem and tentorial ring: technique and clinical experience. Neurosurgery.

[16] Ammerman JM, Lonser RR, Oldfield EH (2005). Posterior subtemporal transtentorial approach to intraparenchymal lesions of the anteromedial region of the superior cerebellum. J Neurosurg.

[17] Moshel YA, Parker EC, Kelly PJ (2009). Occipital transtentorial approach to the precentral cerebellar fissure and posterior incisural space. Neurosurgery.

[18] Spetzler RF, Daspit CP, Pappas CT (1992). The combined supra- and infratentorial approach for lesions of the petrous and clival regions: experience with 46 cases. J Neurosurg.

[19] Peters DR, VanHorn T, Karimian B, Wait SD (2023). Occipital interhemispheric transtentorial approach in pediatric patients for lesions of the superomedial cerebellum: operative findings and results. Childs Nerv Syst.

[20] Samii M, Carvalho GA, Tatagiba M, Matthies C, Vorkapic P (1996). Meningiomas of the tentorial notch: surgical anatomy and management. J Neurosurg.

[21] Katyal A, Jadhav A, Katyal A (2021). Occipital transtentorial approach for pineal region lesions: addressing the controversies in conventional teaching. Surg Neurol Int.

[22] Mottolese C, Szathmari A, Ricci-Franchi AC, Beuriat PA, Grassiot B (2015). The sub-occipital transtentorial approach revisited base on our own experience. Neurochirurgie.

[23] Beuriat PA, Szathmari A, Di Rocco F, Mottolese C (2023). The sub-occipital transtentorial approach for pineal region tumors: how I do it. Acta Neurochir (Wien).

[24] Shepard MJ, Haider AS, Prabhu SS (2022). Long term outcomes following surgery for pineal region tumors. J Neurooncol.

[25] Sun Q, Zhao X, Gandhi S (2019). Quantitative analysis of ipsilateral and contralateral supracerebellar infratentorial and occipital transtentorial approaches to the cisternal pulvinar: laboratory anatomical investigation. J Neurosurg.

[26] Nazzaro JM, Shults WT, Neuwelt EA (1992). Neuro-ophthalmological function of patients with pineal region tumors approached transtentorially in the semisitting position. J Neurosurg.

[27] Kurokawa Y, Uede T, Hashi K (1999). Operative approach to mediosuperior cerebellar tumors: occipital interhemispheric transtentorial approach. Surg Neurol.

[28] Matsushima T, Suzuki SO, Fukui M, Rhoton AL Jr, de Oliveira E, Ono M (1989). Microsurgical anatomy of the tentorial sinuses. J Neurosurg.

[29] Patel PG, Cohen-Gadol AA, Mercier P, Boop FA, Klimo P Jr (2017). The posterior transcallosal approach to the pineal region and posterior third ventricle: intervenous and paravenous variants. Oper Neurosurg (Hagerstown).

